# Behaviour Indicators of Animal Welfare in Purebred and Crossbred Yearling Beef Reared in Optimal Environmental Conditions

**DOI:** 10.3390/ani14050712

**Published:** 2024-02-24

**Authors:** Alessandra Marzano, Fabio Correddu, Mondina Francesca Lunesu, Elias Zgheib, Anna Nudda, Giuseppe Pulina

**Affiliations:** Dipartimento di Agraria, University of Sassari, Viale Italia 39/A, 07100 Sassari, Italy; amarzano@uniss.it (A.M.); fcorreddu@uniss.it (F.C.); e_zgheib@outlook.com (E.Z.); anudda@uniss.it (A.N.); gpulina@uniss.it (G.P.)

**Keywords:** animal welfare, behaviour, yearling beef, purebred, crossbred, Classyfarm^®^

## Abstract

**Simple Summary:**

Behaviour indicators of animal welfare were studied in yearling beef belonging to different genetic types (Limousines, Sardo-Bruna (SRB), and crossbred Limousine × Sardo-Bruna) and sex and reared in the same “optimal” environmental conditions, as prescribed in the Classyfarm^®^ manual. Two trained operators evaluated behavioural activities by the scan and focus samplings and feeding behaviour by evaluating video recording. Behaviour indicators of animal welfare did not evidence substantial differences among animals of different genetic types or sexes reared in the same “optimal” environmental conditions, except for some indicators which were mostly expressed in females and in SRB yearling beef. Female beef and the autochthon’s cattle breed of Sardinia, although typically hardy, showed a wide behavioural repertoire, suggesting a high ability to cope with the environment.

**Abstract:**

The aim of this study was to monitor the behaviour of purebred and crossbred beef cattle reared in the same optimal environmental conditions according to Classyfarm^®^. Thirty-yearling beef 11.5 months old, including 10 Limousines (LMS), 10 Sardo-Bruna (SRB), and 10 crossbred Limousine × Sardo-Bruna (LMS × SRB), balanced for sex and body weight, were used. Animals were evaluated for five months by two trained operators by SCAN (“sternal resting”, “lateral resting”, “ central or peripheral position in the pen”, standing”, “walking”, “feeding”, “drinking”, and “ruminating) and FOCUS (“displacement for space”, “displacement for feed or water”, “play-fighting”, “self-grooming”, “allo-grooming”, “stereotyping”, and “mounting”) protocols. Feeding behaviour was monitored by a CCTV system. The application of the SCAN sampling evidenced that SRB animals preferred the “standing” activity over the LMS animals, while the LMS × SRB did not differ from them. The “standing” and “ ruminating “activities were observed mostly in females than males (*p* < 0.05). For behaviour parameters assessed by the FOCUS methodology, the n-events of “allo-grooming” were higher (*p* < 0.05) in SRB than in LMS and LMS × SRB genetic types. Males showed higher (*p* < 0.05) n-events than females for “play-fighting”. For feeding behaviour, the “eating concentrate” activity (expressed as n-events) was higher (*p* < 0.05) in SRB than LMS × SRB and LMS being intermediate (*p* < 0.05). The duration of “eating concentrate” (expressed in minutes) was higher (*p* < 0.05) in females than males. In conclusion, behaviour indicators of animal welfare did not evidence substantial differences among genetic types and between sexes reared in the same “optimal” environmental conditions. Female beef and the autochthon’s cattle breed of Sardinia, although typically hardy, showed a wide behavioural repertoire.

## 1. Introduction

Within society, animal welfare is subject to a plethora of different ethical, economic, and political viewpoints. Thus, animal welfare can mean different things to different people [1]. Over the years, the concept of animal welfare has changed because of the evolution of the human-animal relationship and the consideration of animals as more conscious beings [2]. When an individual of any species is said to have good welfare, this usually means that they have high levels of pleasure, happiness, contentment, control of interactions with the environment, or opportunities to use their abilities [3]. Then, animal welfare cannot only be the absence of negative experiences or diseases but is a broader concept of good psychophysical quality of life in animals [4]. The mere consideration of adequate environmental conditions or a good health status does not guarantee good animal welfare, but at the same time, the improvement of environmental conditions can improve the health status and the appropriate expression of the behaviour repertoire of the animals. Appropriate behaviour means the chance to exhibit species-specific behaviour, but what does it mean for cattle? The normal behaviour of cattle in an extensive system has a wide behavioural repertoire, but cattle spend a large proportion of their time (90–95% of an animal’s day) in three main behaviours: grazing, ruminating, and resting [5]. A way to valorise farming systems and recognise animal welfare could be to find and measure positive indicators of species-specific behavioural welfare in semi-intensive and intensive farming systems. In particular, the positive indicators of animal welfare were grouped by Napolitano et al. [6] in (a) playful behaviour, (b) synchronicity, (c) positive social behaviour, (d) self- and allo-grooming, scratching and other behaviours. Social positive behaviour includes all behaviours that create a state of well-being for both the performer and the receiver, such as allo-grooming. Napolitano et al. [6] attributed social grooming as a solution for the animal to fight monotony in farming (especially in intensive farming) and found that opioids are produced during allogrooming between animals, acting as a self-narcotic, a remedy for uncomfortable conditions. A final index is self-grooming (self-licking), scratching and other similar behaviours. Unsuitable flooring and inadequate housing conditions affect the expression of these behaviours. Body size and the environment can influence these social behaviours, especially for grooming: males tend to carry out less self-grooming than females, probably due to the larger body dimensions [7]. Another positive indicator of welfare is the decubitus position of the animal [8]: sternal recumbency, with the head resting on the side, and lateral decubitus, with the limbs well extended, are the most comfortable and are adopted when the animal is fully at ease with its surroundings [6].

Different methodologies have been proposed to investigate animal behaviour, especially in primates. Two methods of direct observations ex-vivo, scan sampling and focal sampling, are widely used to collect information about behaviour, respectively, in instantaneous and continuous observations [9,10,11]. Continuous sampling or short scan sampling intervals were validated on pastured lambs [12]. Scan sampling and video recordings were used to assess behaviour and to understand the different eating behaviours of dairy cows [13] and Holstein bulls [14].

Another tool for assessing animal welfare is the examination of the neuroendocrine system through the analysis of serotonin and cortisol in blood, saliva, or urine, although the blood or saliva sampling procedures themselves may affect cortisol levels and may not allow the distinction between acute or chronic cortisolemia [15]. Blood oxytocin can represent an indicator of social wellness because of its implication in neurotransmitter assets of good inter-animal relationships. Oxytocin can socially improve animal welfare by improving grooming behaviour, creating bonds, and stabilising herd relationships [16].

As animal welfare is essentially how each individual responds to the environment, there may be behavioural differences among animals placed in the same environment due to different genetic types. On the other hand, different breeds are a human construct [17], and these have been selected to respond to specific demands of diverse environments. In other words, it cannot be taken for granted that an optimal environmental condition for one breed will be optimal for another. In fact, there is a difference in behaviour patterns between genotypes selected for grazing or for stall-breeding, even within the same breed, as demonstrated by Horan et al. [18] in US Holstein Friesian and New Zealand Friesian. However, the only way to study animal welfare through behaviour is to place different breeds under the same conditions deemed optimal according to a recognised standard.

Classyfarm^®^ [19] is an Animal Welfare evaluation standard protocol adopted by the Italian Ministry of Health through a platform of evaluation criteria and levels with the purpose of preventing antibiotic resistance pathologies, identifying business risks and biosecurity, to monitor and analyse the conditions of farms and make all the necessary improvements to comply with the European legislation on Animal Health Law and Official controls [20]. Classyfarm^®^ is inspired by the “Five freedoms” of Brambell [21], and the protocol helps to assess animal welfare based on three main levels: Management (Caretaker level), Structures and Equipment (Environmental level) and Animal-based measures (ABMs), the same evaluation criteria are applied to all these levels: “Good nutrition”; “Adequate rearing conditions”; “Good health”; “Appropriate behaviour”. The final evaluation categories are: “not adequate”, “adequate”, and “optimal”. Studies on pig [22] and beef cattle [23] have shown that the application of the Classyfarm^®^ standard is able to verify welfare and the related improved production.

Then, the hypotheses tested in this study were: (1) to verify animal welfare conditions in a ClassyFarm standard-compliant barn as a function of cattle genetic type (local breed, beef breed and crossbred) and sex; (2) to apply two survey sampling methods, Focus and Scan, to study welfare behaviour and verify the differences between them in the evaluation of animal status.

## 2. Materials and Methods

### 2.1. Farm Management

The experiment was carried out in a commercial beef farm equipped with a slaughterhouse located in the centre of Sardinia.

The experiment was approved by the Ethics Committee (O.P.B.A) of the University of Sassari (Prot. n. 58790 21 May 2021) and was compliant with the EU Directive 2010/63/EU for animal experiments.

A total of 30 yearling beef (age: 11.5 ± 2.8 months) balanced for sex and body weight (BW: 400.7 ± 53.0 kg) were used. The animals came from other farms, were transferred to the commercial farm for the fattening phase after 9 months from birth, and were enrolled in the study after the quarantine and acclimatisation period. Among them, 10 were from Sardo-Bruna (SRB; autochthons cattle breed of Sardinia region) purebred (5 males and 5 females), 10 were from Limousine (LMS) purebred (5 males and 5 females), and 10 were from Limousine × Sardo-Bruna (LMS × SRB) crossbred (5 males and 5 females).

Animals were allocated to 6 pens of 45 m^2^ with 5 yearling beef per pen, with a space of 9 m^2^/head, with indoor and outdoor paddocks (semi-intensive system), which allowed optimal freedom, as required by the Classyfarm^®^ (Parma, Italy) system [19]. Animals from each genetic type and sex were housed in a single pen. They were monitored for 150 days, from the beginning of August to the end of December 2021.

The pens were equipped with a pergola that covered half of their length, guaranteeing them good shelter. The external feeders allowed access to the diet for all the animals at the same time. The ration, based on hay and concentrate, was administered once a day ad libitum. Troughs were placed every 5-yearling beef, with constant and continuous drinking water. Ventilation was natural, but in the warmer months, horizontal flow fans were used. To evaluate the degree of thermal comfort of the animals, data on environmental temperature and humidity were recorded.

The levels of gaseous ammonia were measured with a specific sensor for NH_3_ (Radiello^®^, Parma, Italy) and the Ammonia ions (NH_4_^+^) between September and December for a total of 10 days of sampling. NH_4_^+^ were quantified by visible spectrometry as indophenol according to Istituti Clinici Scientifici Maugeri guidelines https://radiello.com/english/ammonia_en.htm (accessed on 10 December 2023). Briefly, at a basic buffered pH, the ammonium ion reacts with phenol and sodium hypochlorite, with pentacyanonitrosylferrate catalysis, to form indophenol. The reaction product is intensely coloured blue, and its absorbance is measured at 635 nm. The recommended analytical procedure includes desorption with high-purity deionised water and UV/Vis determination after reaction with phenol, pentacyanonitrosylferrate and sodium hypochlorite in a buffered solution.

Assessments of good health status and appropriate species-specific behaviour were carried out by two trained observers during the experimental period. In the same period, a quantitative evaluation of the feeding behaviour was made by video recordings.

The optimal nutritional status was guaranteed by an ad libitum diet composed of 25% oat straw and 75% commercial concentrate formulated to meet the protein and energy requirements of the animal. The evolution of animal body reserves was evaluated by a monthly measurement of animal body weight (BW).

At the end of the trial, animals were slaughtered during normal farm activity.

### 2.2. Species-Specific Behaviour Evaluations

#### 2.2.1. Ex-Vivo Behaviour Observations

The ex-vivo behaviour animal observations were simultaneously carried out by two trained observers, according to Table 1, using SCAN and FOCUS samplings. Observations were compared between trainees for three control sampling days, and the interobserver reliability agreement was 95%.

The SCAN sampling was applied, observing each pen instantaneously for 5 min and repeated 4 times with an interval of 45 min, one day/month. A checklist was used to assess the following individual behaviour parameters classified as posture or activity. The type of posture was identified as: “sternal resting”, “lateral resting”, “peripheral position”, and “central position”. The type of activity was detected as: “standing”, “walking”, “feeding”, “drinking”, and “ruminating”. Data on SCAN sampling recorded by each observer (during each single scan sampling) were averaged, expressed as a percentage (%) of animals/pen and then used as a variable in the statistical model.

The FOCUS sampling was applied, observing each pen continuously for 15 min and repeating the observation 5 times with an interval of 45 min, one day/month. A checklist was used to assess the following individual behaviour parameters: “head shots”, “displacement for feed or water”, “displacement for space”, “play-fighting”, “self-grooming”, “allo-grooming”, “stereotyping”, and “mounting”. Data of FOCUS sampling recorded by each observer (during each single focus sampling) were averaged, then summed up within each day, and expressed in n-events.

#### 2.2.2. Behaviour Observations from Video-Recordings

All pens were filmed by 6 video cameras (CCTV system, 1 video camera/pen). They were installed to see the images from the feeder to about half of their length so that the animals could be seen feeding from the front. The behaviour of the yearling beef was analysed by observing 60 video recordings for a standard time of 45 min (the duration of some exceeded 60 min); each box was observed by four videos/month, corresponding to 4 different moments of the same day. All data from video recordings and observations were summed up by pen within each month. Video observation was directed to assess the number of events considered to be “n-events” in which the animals approached the meal to “eating hay” or “eating concentrate”, “competing at the feeder”, “ruminating”, and “resting”.

For all activities, “eating hay” or “eating concentrate”, “competing at the feeder”, “ruminating”, and “resting”, the duration was measured in minutes. The duration “at the feeder”, useful for frequency “at the feeder” was calculated by summing the time for “eating hay” and for “eating concentrate”.

The frequencies of “eating hay” or “eating concentrate” and the “frequency “at the feeder” were calculated by the ratio between Σ n-events/duration.

### 2.3. Blood Oxytocin Determinations

At the beginning of the experimental period, during the routine inspection of the animals by the farm veterinarian, blood samples were taken from 20 animals. The blood samples were taken for health evaluation and also during the slaughter of each animal. Blood was collected in vacutainer tubes with anticoagulant (Li-heparin), maintained at 4 °C, and centrifuged at 3000 rpm for 10 min to separate the plasma portion. Plasma samples were collected in 2 mL centrifuge tubes and stored at −80 °C until oxytocin analysis. Bovine plasma samples were thawed and kept in a thermal shaker at 37 °C for 1 h prior to analysis. The ELISA (Enzyme-Linked Immunosorbent Assay) technique, an enzyme-linked immunosorbent assay, was used for the oxytocin assay. The oxytocin concentration, expressed in pg/mL, was determined with a bovine oxytocin ELISA kit (Oxtc-BOEB1280 Assay Genie, Dublin, Ireland).

### 2.4. Statistical Analysis

Data on behaviour parameters assessed by the SCAN and FOCUS sampling and by video recording were analysed by a PROC MIXED procedure of SAS^®^ (SAS Institute Inc., Cary, NC, USA), with the following linear model:

y*_ijklm_* = *μ* + GT*_i_* + Sex*_j_* + (GT × Sex)*_ij_* + Time*_k_* + Box*_l_* + *e_ijklm_*

where *y* is the considered trait; *μ*, is the overall mean; GT is the fixed effect of the genetic type (Limousine purebred, Sarda-Bruno purebred, and Limousine × Sardo-Bruno crossbred); Sex is the fixed effect of the sex (males, females); GT × Sex is the fixed effect of the interaction between genetic type and sex; Time is the fixed effect of sampling month (4 levels for the FOCUS and SCAN measurements, 3 levels for the video recordings); box, is the random effect of the box; and *e* is the residual term.

Data on blood oxytocin were analysed using the PROC GLM procedure of SAS^®^ (SAS Institute Inc.) to test any differences among genetic type (LMS, LMS × SRB, and SRB), sex, and their interaction.

Differences among means were declared significant when *p* < 0.05.

## 3. Results

All animals were in good health status throughout the experiment, as assessed by the farm’s veterinarian.

During the experimental period, all animals were in good nutritional status and had good housing with ammonia levels that never exceeded 10 ppm. Therefore, the rearing condition was ranked as the “optimal” category in accordance with Classyfarm^®^.

### 3.1. Ex-Vivo Behaviour Observations by SCAN Sampling

The behaviour parameters for posture and activities (expressed in % of animals), assessed by SCAN sampling, are presented in Table 2. The interaction genetic type × sex was significantly different for all “posture” variables (*p* < 0.05). Females of SRB had lower values of sternal resting compared to the males of the same genetic type and to the other animals. Males of the crossbred showed higher values for “lateral resting” and “peripheral position” than the females of the crossbred and compared to the other animals, regardless of sex; a similar result, but of the opposite sign, was found for the “central position”.

Regarding the “activities” variables, only “standing” and “ruminating” were influenced by the considered factors. Specifically, “standing” was influenced by the genetic type (*p* < 0.05) and sex (*p* < 0.05), whereas “ruminating” was influenced by the sex (*p* < 0.05). The SRB showed a higher value of “standing” than LMS, with the crossbred not differing from them. Independent of the genetic type, the females had higher values of “standing” and “ruminating” than males. These variables were not influenced by the genetic type × sex interaction.

### 3.2. Ex-Vivo Behavioural Observations by FOCUS Sampling

No significant differences were observed between genetic type and sex for behaviour parameters assessed by the FOCUS sampling, except for “allo-grooming” and “play-fighting”, as shown in Table 3. The n-events of “allo-grooming” were higher in SRB than in LMS and LMS × SRB genetic types (*p* < 0.05). In addition, males showed higher n-events for “play-fighting” than females (*p* < 0.05).

### 3.3. Behaviour Parameters from Video Recording Observations

The video recording observations are presented in Table 4.

The genetic type influenced “eating concentrate” (expressed as n-events) and the frequency of “eating hay” (n-events/minutes), with SRB presenting higher values than crossbred and LSM being intermediate (*p* < 0.05). The duration of “eating concentrate” (expressed in minutes) was influenced by sex, with females having higher values than males (*p* < 0.05). None of the parameters of the video recording was influenced by the genetic type × sex interaction.

**Figure 1:**
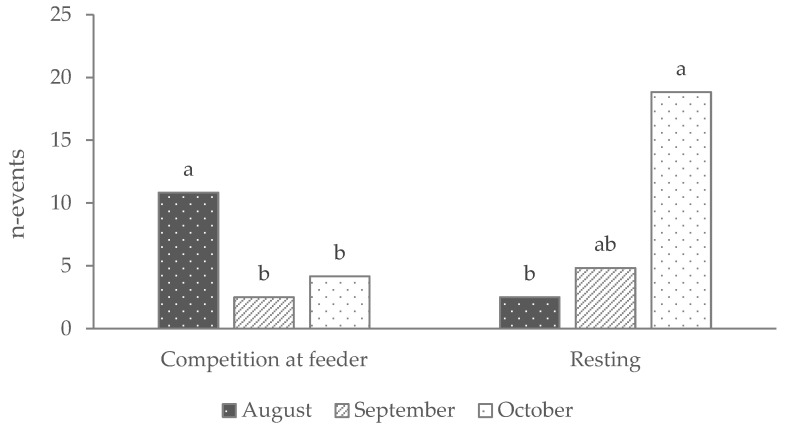
“Competition at feeder” and “Resting” (expressed as n-events) evaluated during the three months of video recording observations. The effect of time was significant (*p* < 0.01). Different superscripts indicate significant differences among means.

**Figure 2:**
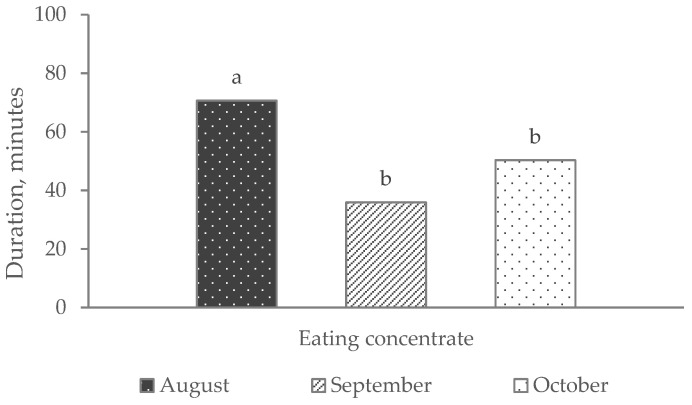
“Eating concentrate” (expressed as minutes) evaluated during the three months of video recording observations. The effect of time was significant (*p* < 0.01). Different superscripts indicate significant differences among means.

N-events of “competition at feeder” and “resting” (Figure 1) and the duration of “eating concentrate” (Figure 2) were significantly influenced by the time (month of video recording). The n-events of “eating concentrate” did not reach the statistically significant threshold. The “competition at feeder” (n-events) and the duration of “eating concentrate” (minutes) were higher in August than in September and October (*p* < 0.05), whereas “resting” (n-events) was higher in October than in August (*p* < 0.05) and September (numerically, *p* < 0.10).

### 3.4. Oxytocin Determinations in Yearling Beef Plasma

Plasmatic oxytocin did not show differences among genetic types, even if the SRB genetic type showed a numerically higher concentration of oxytocin compared to the other animals (Table 5). Males showed significantly higher values at slaughter than females.

## 4. Discussion

Appropriate behaviour, together with health status and herd management, can provide a more complete and holistic view of animal welfare. In the last decades, there has been a shift in Western countries to consider so-called positive animal welfare, which focuses on behaviour expression of what constitutes a good animal life [24]. As found in this study, the availability of space and the optimal farm management, according to the Classyfarm^®^ checklists, can affect the expression of the appropriate behaviour of the yearling beef. Welfare issues have to be resolved in the context of the farming system, not considering only the experiences of the animal; on the other hand, environmental shifts and temperature conditions could result in greater energetic needs and ultimately impact cattle lying behaviour and feeding behaviour [25]. So, it is important to find indicators that confirm animal welfare together with optimal rearing conditions.

In our study, the most relevant differences concerning the general activities were for resting and standing: the SCAN sampling showed a greater attitude of animals to rest than to stand, except for SRB and females, which showed a greater attitude to stand. In addition, lateral resting seemed to be a more frequent posture for crossbred male beef. Napolitano et al. [6] highlighted that resting alone cannot be interpreted as a sign of well-being since it is essential for the animal, but it is also important to consider the position adopted. The sternal resting, with the head resting on the side, is one of the most comfortable positions for cattle; it can, therefore, be understood as a sign of comfort and trust of the animal towards the environment [6]. The position inside the pen could also be an interesting item to observe because it indicates the ability to dominate the environment (central) or to inhabit it uncertainly (peripheral). In our study, we observed a different behaviour of LMS × SRB females that preferred the central position more than the other genetic types; it could express a way of adaptation in the environment, albeit semi-confined. If there is no suitable resting area, the animals will tend to spend most of their time standing; this would lead to repercussions both on behaviour and physical level, with an increase in problems with the limbs, mainly with the hoofs [26]. On the other hand, Jensen and Kyhn [26] found that the duration and frequency of lying behaviour and the time spent standing without eating appear to be probable behavioural indicators of cow comfort.

Grooming is frequent in cattle, and it is important for maintaining the animal’s hygiene and for expressing sociability. This behaviour also responds to various physiological and environmental factors, suggesting a possible link to animal comfort and well-being [27]. The SRB was also found to be more involved in social activities (FOCUS sampling) such as “allo-grooming”, behaviours probably used to reduce stress, both socially and individually, as yet found in literature [6].

Concerning general and social behaviours, the SRB beefs have probably found a way of adapting to the environment and to the new semi-confined condition, thus a strategy to reduce rearing stress.

Embryology studies have also shown that the same stress (oxidative stress) can differentially impact males’ and females’ early embryos depending on the culture medium of the stress inducer [28]. Males calves suffer more significant consequences of weaning than females [29]. The differences between males and females showed in our study (“standing” and “ruminating” more expressed by females) did not confirm these previous observations. Concerning the social behaviour of males, the optimal density condition allowed freedom of movement, and so the males exhibited their behaviour repertoire of playful fighting, as widely confirmed in the literature [26].

Feeding behaviour traits are a mirror of some animal performance metrics and an interesting indicator of behaviour animal welfare. As demonstrated by Kelly et al. [30], animals experiencing a state of well-being tend to take a meal over a longer time and with a lower frequency to increase the metabolisable energy content. The observation of feeding behaviour by means of video camera recordings was aimed at carrying out a preliminary study to understand how yearling beef of different breeds behave and to find some indicators of behaviour animal welfare. It would be interesting to evaluate if local breeds, as SRB, reared in a semi-confined system, could have different eating behaviours than the other genetic types used to more intensive farming systems. To the best of our knowledge, there are no studies in literature concerning the comparison of feeding behaviour among beef of different breeds. Our study evidenced that there are no substantial differences among LMS, LMS × SRB, and SRB genetic types for feeding behaviour. We only observed the higher n-events of concentrate eating; indeed, it is well known that increasing concentrate intake is a strategy for cattle to reduce stress for higher temperatures, as concentrate digestion is less calorific than hay digestion [31]. The hay-eating frequency in SRB compared to LMS × SRB could probably be explained by the habit of the SRB genetic type to graze more fibrous grass.

The monthly comparison of behaviour parameters showed that in August, all yearling beef displayed a higher eating concentrate time; this was expected due to the higher temperatures of August. The need to eat more concentrate could be the reason for the increased competition at the feeder observed in August compared to later months. The higher duration of eating concentrate found in the females could probably be associated with the ability of females to be more resilient than males. On the other hand, the increase in resting events observed in October compared to the previous months could be a confirmation of the well-being and ability of all animals to adapt to the environment: when temperatures were more comfortable, animals rested more. Then, also regarding feeding behaviour, we can assume that the optimal environmental condition favoured a better adaptation of animals to environmental challenges.

The lack of significant differences in oxytocin levels among genetic types was previously observed by Chen et al. [32,33], who found high variability of serum oxytocin levels among animals. It is worth noting that the highest numerical oxytocin values registered in SRB beef suggest, together with other behaviour items, the ability of this breed to maintain a state of superior well-being despite the novelty of the environment. In fact, oxytocin may be involved in habituation to a novel environment in cattle, and it may reflect an association between oxytocin and a “familiarization-habituation response” [34,35]. The level of oxytocin at slaughtering confirms the absence of differences between genetic types. Higher oxytocin concentration in males than females at slaughtering could be due to a higher adaptive capacity during pre-slaughter stress. In this perspective, the relationship between behaviour and oxytocin should be better explored. Thus, further investigations are needed.

## 5. Conclusions

This study confirms that the environmental and structural conditions of farming in the “optimal” category, as prescribed in the Classyfarm^®^ manual, can positively contribute to animal welfare. From the present study, behaviour indicators of animal welfare did not evidence substantial differences among genetic types and between sexes reared in the same “optimal” environmental conditions, except for some indicators which were mostly expressed in females and in SRB yearling beef. The autochthons cattle breed of Sardinia, typically hardy, showed a wide behavioural repertoire, suggesting a high ability to cope with the environment.

## Figures and Tables

**Table 1:** The definition of behavioural activities which was agreed upon between the two observers.

Item ^1^	Definition
SCAN sampling, each pen instantaneous for 5 min, every 45 min for general activities	
Sternal resting	When an animal was lying down on the sternum
Lateral resting	When an animal was lying down laterally along the body
Peripheral Position	When an animal occupied a peripheral position in the pen
Central position	When an animal occupied a central position in the pen
Standing	When an animal was standing on 4 legs
Walking	When an animal walked around the pen
Feeding	When an animal approached the feeder and consumed the meal
Drinking	When an animal approached the trough and consumed water
FOCUS sampling, each pen continuous for 15 min, every 45 min for social activities	
Head shots	When an animal has hit the wall or fence with its head
Displacement F/W	When an animal competed with another animal for a meal or water
Displacement space	When an animal would sneak between 2 animals to gain space
Play-fighting	When two animals engaged in fast galloping, interrupted by sudden changes of direction, arching, kicking the hind legs, rotations, and twists of the body, without an intent to defend or fight
Self-grooming	When an animal licked its own body in a non-stereotyped way, scratching with a hind limb or against fixture
Allo-grooming	When an animal licked a neighbor along the body
Stereotipy	Tongue rolling or biting on bars or other sites in the barn
Mounts	When an animal jumped on the back of another animal

^1^ F = Feed; W = Water.

**Table 2:** Behavioural parameters assessed by the SCAN sampling (expressed as a percentage of animals) for the genetic types of Limousine (LMS), Sardo-Bruna (SRB), and their crossbred (LMS × SRB).

Item		Genetic Type			Sex Mean	SEM ^1^	*p*-Value ^2^			
	Sex	LMS	LMS × SRB	SRB			GT	Sex	GT × Sex	Time
Posture, % of animals										
Sternal Resting	Females	58.0 ^a^	40.7 ^ab^	27.3 ^b^		3.13	0.044	0.015	0.020	<0.0001
	Males	50.7 ^ab^	59.3 ^a^	52.0 ^a^						
	GT mean									
Lateral Resting	Females	2.0 ^b^	2.7 ^b^	0.0 ^b^		0.75	<0.0001	0.105	0.003	0.003
	Males	0.0 ^b^	10.7 ^a^	0.0 ^b^						
	GT mean									
Peripheral position	Females	54.0 ^b^	36.7 ^b^	47.3 ^b^		3.26	0.202	0.016	<0.0001	<0.0001
	Males	43.3 ^b^	78.0 ^a^	50.0 ^b^						
	GT mean									
Central position	Females	40.0 ^ab^	56.7 ^a^	54.0 ^a^		3.34	0.128	0.020	0.001	<0.0001
	Males	50.0 ^a^	20.0 ^b^	46.7 ^a^						
	GT mean									
Activities, % of animals										
Standing	Females	38.7	50.0	61.3	50.0 ^a^	3.31	0.035	0.044	0.219	<0.0001
	Males	40.7	35.3	46.0	40.7 ^b^					
	GT mean	39.7 ^b^	42.7 ^ab^	53.7 ^a^						
Walking	Females	1.3	2.7	5.3	3.1	0.75	0.225	0.778	0.358	<0.0001
	Males	4.0	0.0	4.0	2.7					
	GT mean	2.7	1.3	4.7						
Feeding	Females	18.7	23.3	24.0	22.0	1.85	0.546	0.293	0.144	0.039
	Males	24.0	11.3	19.3	18.2					
	GT mean	21.3	17.3	21.7						
Drinking	Females	4.0	2.0	4.0	3.3	0.65	0.075	0.852	0.377	0.001
	Males	6.0	1.3	2.0	3.1					
	GT mean	5.0	1.7	3.0						
Ruminating	Females	16.0	15.3	8.0	13.1 ^a^	1.42	0.261	0.022	0.601	0.301
	Males	6.7	8.0	5.3	6.7 ^b^					
	GT mean	11.3	11.7	6.7						

^1^ SEM = standard error of the mean, related to the overall mean for each considered variable. ^2^ GT = genetic type; GT × Sex = genetic type × sex interaction. ^a,b^ Different superscripts indicate significant differences among means (*p* < 0.05).

**Table 3:** Behaviour parameters assessed by the FOCUS sampling (expressed as a percentage of animals) for the genetic types Limousine (LMS), Sardo-Bruna (SRB), and their crossbred (LMS × SRB).

Item	Genetic Type			Sex		SEM ^1^	*p*-Value ^2^			
	LMS	LMS × SRB	SRB	Females	Males		GT	Sex	GT × Sex	Time
Head shots	0.38	0.25	0.50	0.08	0.67	0.191	0.839	0.109	0.480	0.067
Displacement for water/feed	2.06	1.00	1.81	2.04	1.21	0.599	0.241	0.125	0.955	<0.0001
Displacement for space	0.06	0.25	0.25	0.21	0.17	0.079	0.299	0.708	0.185	0.001
Play-fighting	1.13	0.50	0.50	0.17 ^b^	1.25 ^a^	0.229	0.354	0.015	0.282	0.364
Allo-grooming	0.94 ^b^	0.81 ^b^	2.44 ^a^	1.38	1.42	0.316	0.032	0.935	0.600	0.029
Self-grooming	3.06	2.00	4.56	2.83	3.58	0.727	0.165	0.482	0.242	0.003
Stereotyping	0.06	0.19	0.25	0.13	0.21	0.065	0.374	0.451	0.048	0.076
Mounting	0.06	0.00	0.81	0.42	0.17	0.210	0.240	0.557	0.577	0.312

^1^ SEM = standard error of the mean. ^2^ GT = genetic type; GT × Sex = genetic type × sex interaction. ^a,b^ Different superscripts indicate significant differences among means (*p* < 0.05).

**Table 4:** Behaviour parameters assessed during three months of video recording observations (expressed in n-events, duration, or frequency) for the genetic types of Limousine (LMS), Sardo-Bruna (SRB), and their crossbred (LMS × SRB).

Item	Genetic Type			Sex		SEM ^1^	*p*-Value ^2^			
	LMS	LMS × SRB	SRB	Females	Males		GT	Sex	GT × Sex	Time
n-events										
Eating concentrate	30.17 ^ab^	18.67 ^b^	38.17 ^a^	30.56	27.44	3.734	0.048	0.587	0.265	0.039
Eating hay	35.67	21.33	40.00	33.00	31.67	3.244	0.054	0.819	0.761	0.206
Competition at feeder	8.67	3.17	5.67	6.11	5.56	1.258	0.051	0.732	0.175	0.004
Resting	12.67	4.83	8.67	11.22	6.22	2.402	0.142	0.119	0.201	0.002
Rumination	4.83	0.67	3.17	3.22	2.56	0.816	0.171	0.697	0.911	0.442
Duration, minutes										
Eating concentrate	42.99	57.60	56.28	58.15 ^a^	46.43 ^b^	4.507	0.070	0.041	0.846	0.001
Eating hay	72.69	65.28	60.30	72.58	59.60	4.232	0.509	0.157	0.254	0.985
Competition at feeder	160.87	122.89	103.63	127.01	131.26	18.786	0.487	0.914	0.323	0.352
Frequency, n-events/minutes										
At feeder	0.50	0.33	1.06	0.50	0.76	0.165	0.197	0.426	0.358	0.484
Eating concentrate	0.75	0.38	0.66	0.55	0.65	0.070	0.068	0.402	0.200	0.420
Eating hay	0.49 ^ab^	0.33 ^b^	0.74 ^a^	0.46	0.58	0.070	0.030	0.265	0.184	0.194

^1^ SEM = standard error of the mean. ^2^ GT = genetic type; GT × Sex = genetic type × sex interaction. ^a,b^ Different superscripts indicate significant differences among means (*p* < 0.05).

**Table 5:** Oxytocin concentration in Limousine (LMS), Sardo-Bruna (SRB) and their crossbred (LMS × SRB) during the growing phase and at slaughter.

Oxytocin, pg/mL	Genetic Type			Sex		SEM ^1^	*p* Value ^2^	
	LMS	LMS × SRB	SRB	Females	Males		GT	Sex
at farm	249.5	201.6	657.6	319.9	419.2	94.78	0.374	0.468
at slaughering	367.9	186.3	368.9	135.0 ^b^	480.4 ^a^	82.40	0.546	0.050

^1^ SEM = standard error of the mean. ^2^ GT = genetic type. ^a,b^ Different superscripts indicate significant differences among means (*p* < 0.05).

## Data Availability

The data presented in this study are available on request from the corresponding author.
